# Molecular diagnostics and therapies for gastrointestinal tumors: a real-world experience

**DOI:** 10.1007/s00432-021-03774-5

**Published:** 2021-08-26

**Authors:** Sabrina Welland, Tiago deCastro, Melanie Bathon, Thomas Christian Wirth, Tanja Reineke-Plaaß, Michael Saborowski, Ulrich Lehmann, Anna Saborowski, Arndt Vogel

**Affiliations:** 1 Department of Gastroenterology, Hepatology and Endocrinology, Hannover Medical School, Carl-Neuberg-Str. 1, 30625 Hannover, Germany; 2 Department of Pathology, Hannover Medical School, Carl-Neuberg-Str. 1, 30625 Hannover, Germany

**Keywords:** Precision oncology, Personalized cancer medicine cholangiocarcinoma, Targeted therapy, Panel sequencing

## Abstract

**Purpose:**

Several targeted agents demonstrated efficacy in early clinical trials for gastrointestinal (GI) cancers, but in many cases, phase-III trials and/or approval by the European Medicines Agency (EMA) are lacking. The primary focus of this study was to assess the regulatory processes associated with use and reimbursement of off-label treatment in precision oncology and to evaluate the benefit of targeted therapy in a real-world population in Germany.

**Methods:**

Our cohort comprises 137 patients with GI cancers and is biased towards cancer entities with a high frequency of known targetable alterations, such as cholangiocarcinoma. Genetic testing was used to identify molecular targets, and therapy response was evaluated based on CT scans.

**Results:**

A molecular target for precision oncology was identified in 53 patients and 43 requests for cost coverage were submitted to health insurance companies. 60% of the requests received approval after initial application and another 7% after appeal. Half of the rejected requests were denied despite ESCAT IA level evidence. The median time between initiation of molecular testing and start of therapy was 75 days. 35 patients received matched targeted therapies (*n* = 28) or, in the case of MSI, immunotherapy (IO) (*n* = 7). We observed a trend in favor of molecular therapy when compared to the immediate prior treatment.

**Conclusion:**

Relevant treatment options were identified by molecular testing in a significant subset of patients. When targeted therapies that lack EMA approval are considered, treatment initiation may be delayed by the duration of the molecular analysis and the regulatory processes.

**Supplementary Information:**

The online version contains supplementary material available at 10.1007/s00432-021-03774-5.

## Introduction

In gastrointestinal (GI) oncology, genetic alterations can serve both as negative, as well as positive predictive biomarkers. For instance, since the concept that *KRAS* mutant colorectal cancer patients do not benefit from the addition of EGFR antibodies to the chemotherapy backbone was introduced, determination of RAS status has become part of the routine diagnostic workup in patients with advanced colorectal cancer (Di Fiore et al. 2007; Karapetis et al. 2008). In contrast, while there is accumulating evidence that microsatellite instable (MSI) GI cancers do not derive significant benefit from perioperative chemotherapy, several studies confirmed a strong positive correlation between MSI high status and response to immunotherapies (Andre et al. 2020; Le et al. 2017b; Pietrantonio et al. 2019; Seymour and Morton 2019; Smyth et al. 2017). Genetic alterations can also create distinct vulnerabilities and serve as targets for precision oncology approaches. With an expanding repertoire of targeted agents and clinical data on precision oncology in solid tumors, molecular diagnostics using high-throughput sequencing technologies are becoming increasingly important and are integrated in routine clinical diagnostics. Especially in higher lines of therapy and in malignancies with limited therapeutic options, panel sequencing can identify molecular targets for therapy and help to ensure, that the full spectrum of clinically meaningful treatment options is offered to patients. However, access to targeted drugs is often hampered by the lack of approval by the European authorities, namely the European Medicines Agency (EMA), and without this approval, treating physicians need to file for cost coverage by the health insurance companies on an individual basis.

In the following, we report our experiences with molecular diagnostics in GI malignancies, outline the formal requirements and temporal processes associated with approval of cost coverage by German health insurance companies, and assess the clinical response we observed in patients that received individualized therapies in our center.

## Results

### Molecular diagnostics in clinical routine

Targeted gene panels cover a distinct set of regions within the genome and serve as powerful and cost-effective tools to identify therapeutically relevant alterations in solid malignancies. The size of the panels varies considerably, ranging from only few genes with direct therapeutic implications, to larger panels that detect also more rare genetic variants, or recurrent alterations without direct therapeutic implications. Between March 2019 and April 2020, 118 patients received tumor-genetic testing via panel sequencing in our GI oncology unit. The most frequently applied panel was the Oncomine Comprehensive Cancer Assay v3 that was performed in-house and covers 161 of the most relevant cancer driver genes based on an amplicon approach (109/118). In contrast, the Foundation One CDx assay is a hybrid capture approach and was performed in a small subset of patients (9/118) by an affiliated external service provider.

**Fig. 1:**
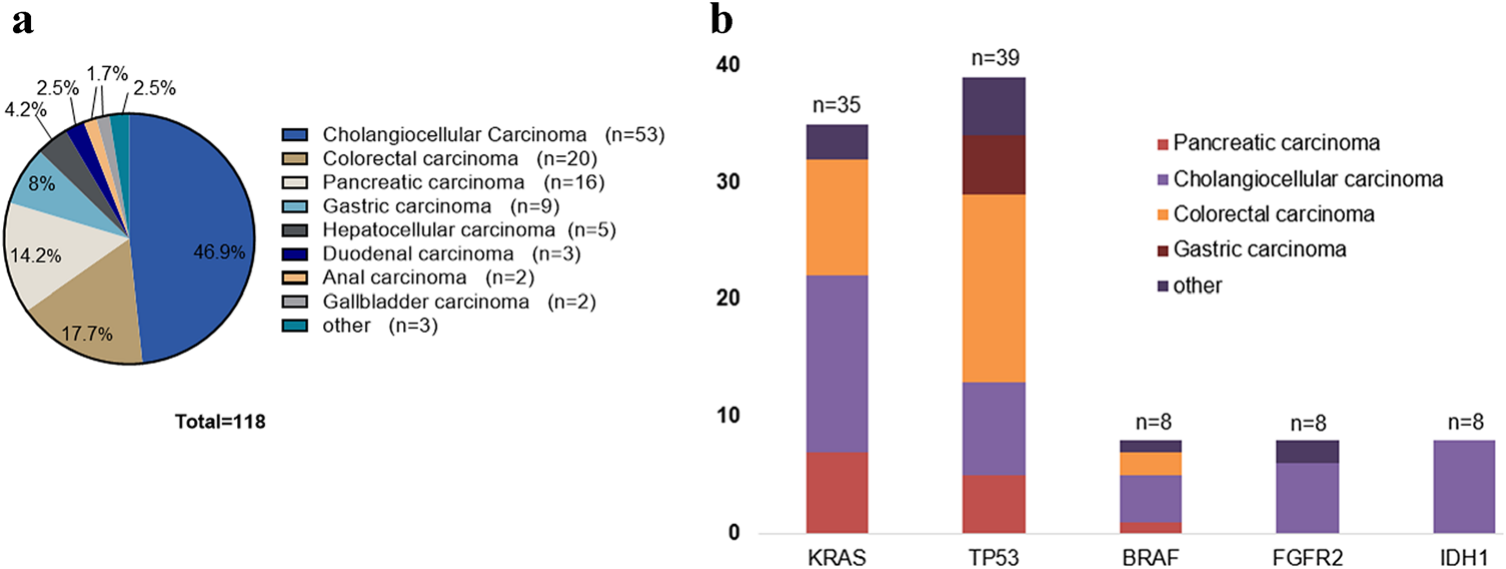
Frequency of tumor types of patients with panel sequencing (n=118) (**a**), most frequent genetic alterations detected in the panel (**b**)

The cohort that received genetic testing beyond the current standards (such as determination of *KRAS* status in left-sided colorectal cancer) was heavily biased towards cholangiocellular carcinomas (48.3%), followed by colon carcinomas (16.9%), pancreatic carcinomas (14.4%) and gastric carcinomas (7.6%) (Fig. 1a). The mean duration from material submission to receipt of the results was 34.5 days, with longer periods often caused by delayed transfer of tumor samples from external pathologies. The quantity or quality of the tissue was insufficient for sequencing in 13 cases, leading to the subsequent exclusion of these samples from the analysis. In five cases, a re-biopsy was performed.

In 101/118 cases at least one genetic alteration was detected, whereas no alterations were reported in 17 samples. Seven out of these 17 cases were re-sequenced using the FoundationOne CDx assay, which led to the detection of at least one alteration in all cases. In two patients, these results were of direct therapeutic significance due to detection of FGFR2 fusions. The most frequently detected genetic alterations were detected in *TP53* (34.5%), followed by *KRAS* (31%), *IDH* (7%), *FGFR2* (7%), *BRAF* (7%) and ERBB2/Her2 (5.3%) (Fig. 1b). The number of mutations varied between one and nine, with an average of 2.78 genetic aberrations per patient. In 22/113 cases, tier I lesions as defined by the ESMO Scale of Clinical Actionability for molecular Targets (ESCAT) were identified [evidence level of ESCAT IA (*n* = 12/29), IB (*n* = 7/29) and IC (*n* = 3/29)] (Mateo et al. 2018) and in seven cases, the evidence reached an ESCAT level of II or III.

### Cost coverage application to the health insurance companies

**Fig. 2:**
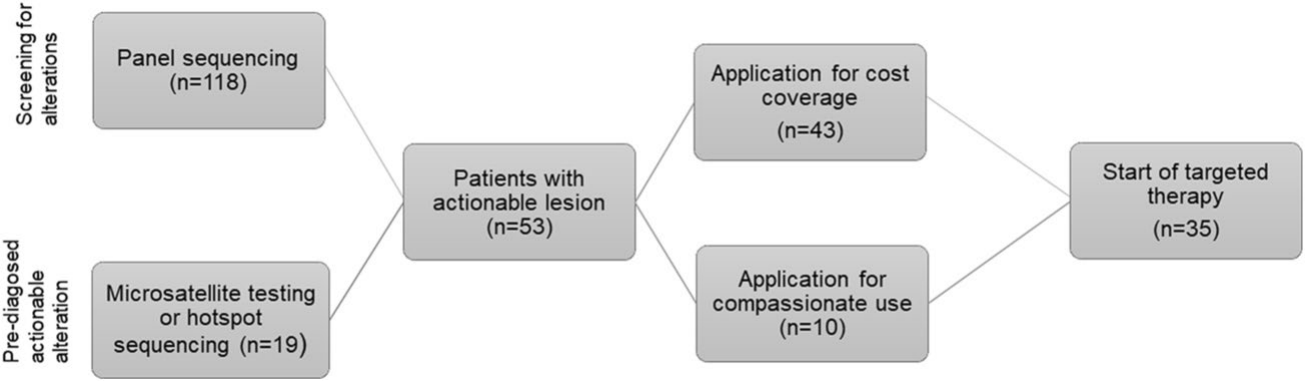
Overview of real-world cohort with screening for actionable alterations, cost coverage application and start of targeted therapy

A cohort of 53 patients with actionable lesions was identified via in-house panel sequencing (*n* = 34) or, especially in patients with colon cancer, by targeted sequencing of hotspot regions (such as *BRAF*) as well as testing for microsatellite stability or PDL-1 expression (*n* = 19) (Fig. 2). Baseline characteristics of all patients are presented in supplementary table 1.

While 43 applications for cost coverage for individualized treatments were submitted to the health insurance companies, the remaining ten patients received an FGFR Inhibitor in the context of a compassionate use program.

**Fig. 3:**
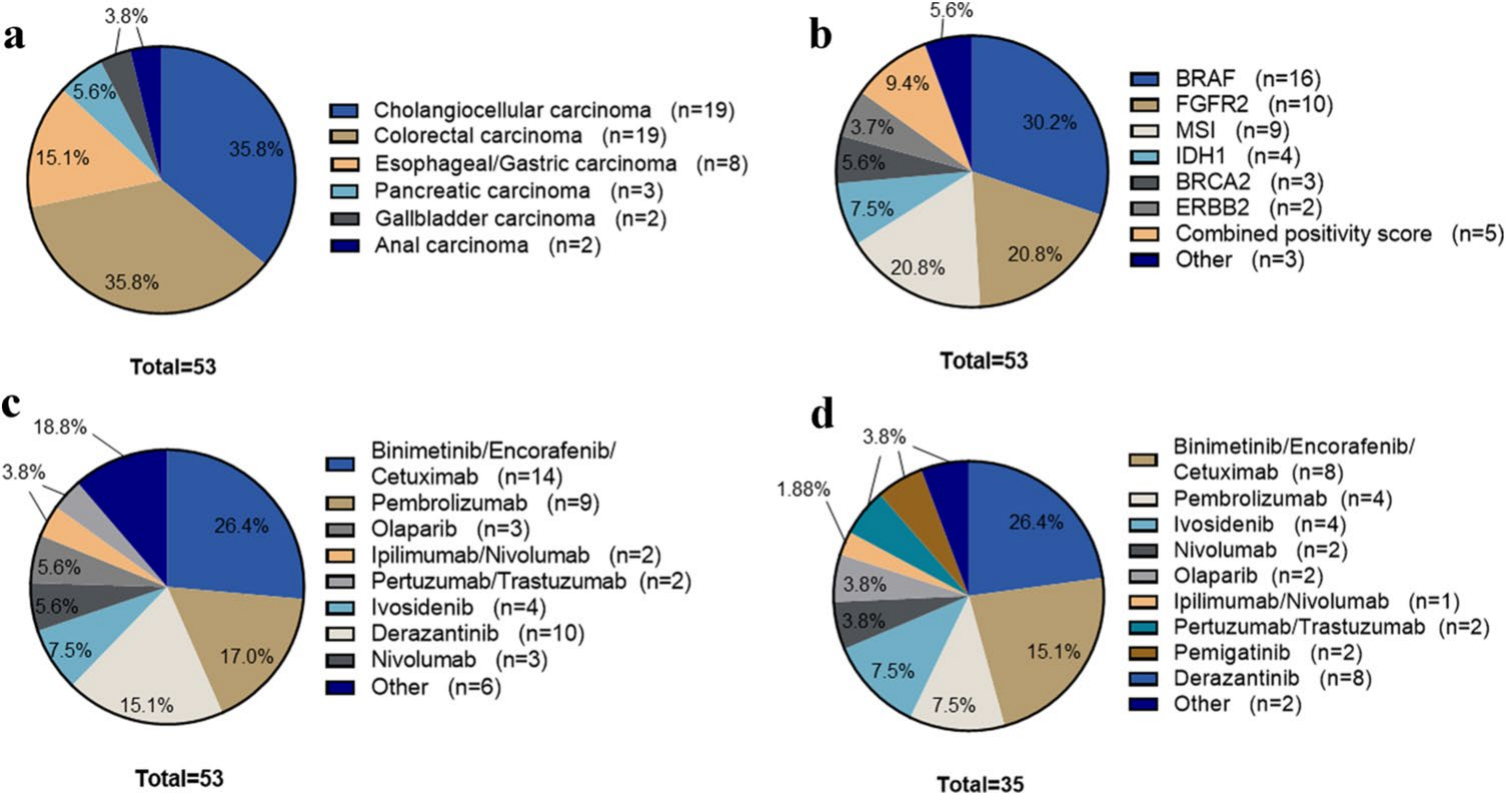
A molecular target was identified in 53 patients. **a** patients with molecular target according to disease entitiy, **b** according to molecular alteration/ biomarker, and **c** according to intended medication. **d** 35 patients were started on molecular therapies as indicated

**Fig. 4:**
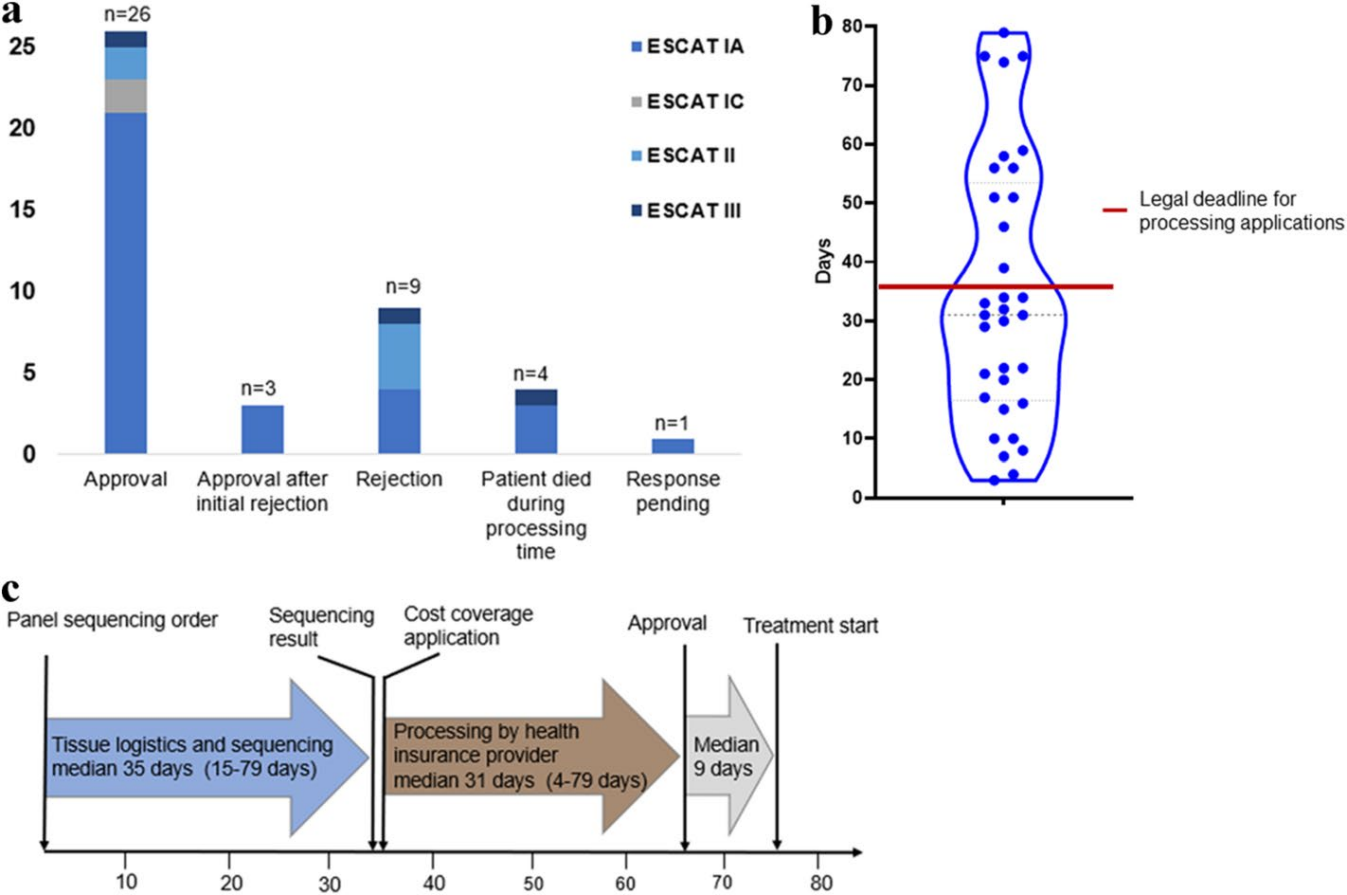
In total, 43 applications for treatment with targeted therapies were filed. **a** responses of the health insurance companies indicating the respective ESCAT levels. **b** processing time per individual application. **c** graphical illustration of the temporal requirements from inititation of panel sequencing to start of treatment

With 36% each, the most frequent disease entities were cholangiocellular carcinoma and colorectal carcinoma (Fig. 3a). In part, patients were heavily pretreated with a median of three prior therapies (mean 2.78). The most frequent actionable lesions were activating *BRAF* mutations (V600E, *n* = 14; D594G, *n* = 2; 30.2%, 14/16 occurred in patients with CRC), activating *FGFR2* mutations (*n* = 2) or FGFR2 fusions (*n* = 8) (18.8%, all of which occurred in patients with CCA), and microsatellite instability/dMMR (17%), as assessed by PCR and immunohistochemistry (Fig. 3b, c). Cost coverage requests were based on proof of concept from phase III (69.8%) and phase II trials (30.2%) (Suppl. Table 2). 79% (*n* = 34) of the approved treatments were categorized as ESCAT level I (IA or IC), 21% of the applications were categorized into a lower ESCAT level. 26 applications (60.5%) were approved by the health insurance companies upon the initial request. In twelve cases (27.9%) the first application was rejected (seven with ESCAT IA and five with a lower ESCAT level). Consequently, seven patients filed an appeal, which was granted in three cases (Fig. 4a). In the rejection letters, an alternative treatment was usually suggested, which, however, we had deemed either less promising or too toxic for the patient. In some cases, recommendations were given that were clearly not indicated, such as the administration of anti-EGFR substances in *KRAS*-mutated colorectal carcinoma.

The timeframe required by the insurance agencies to process and respond to the requests varied profoundly. On average, the median duration from application to first feedback from the health insurance company regarding acceptance or rejection was 31 days (ranging between 4 and 79 days) (Fig. 4b). According to section 13 paragraph 3a Volume V of the German Social Insurance Code, the legal period for processing an application is 3 weeks, with a maximum extension to 5 weeks if a medical expert opinion is required. In 11 of 43 cases (26%) the five-week cutoff was exceeded, which, in some cases, significantly delayed therapy initiation. Seven patients died before (*n* = 4) or shortly after (*n* = 3) receiving confirmation of cost coverage and prior to initiation of the targeted therapy. In total, a median of 75 days elapsed from the time of initiation of molecular diagnostics, to the start of molecular therapy (Fig. 4c). In particular, there were significant delays due to tissue logistics (transfer from external pathologies), and lengthy processing of applications by the health insurance companies. In individual cases, this led to a cumulative delay in therapy initiation of up to 6 months.

### Individualized tumor therapy for gastrointestinal tumors

**Fig. 5:**
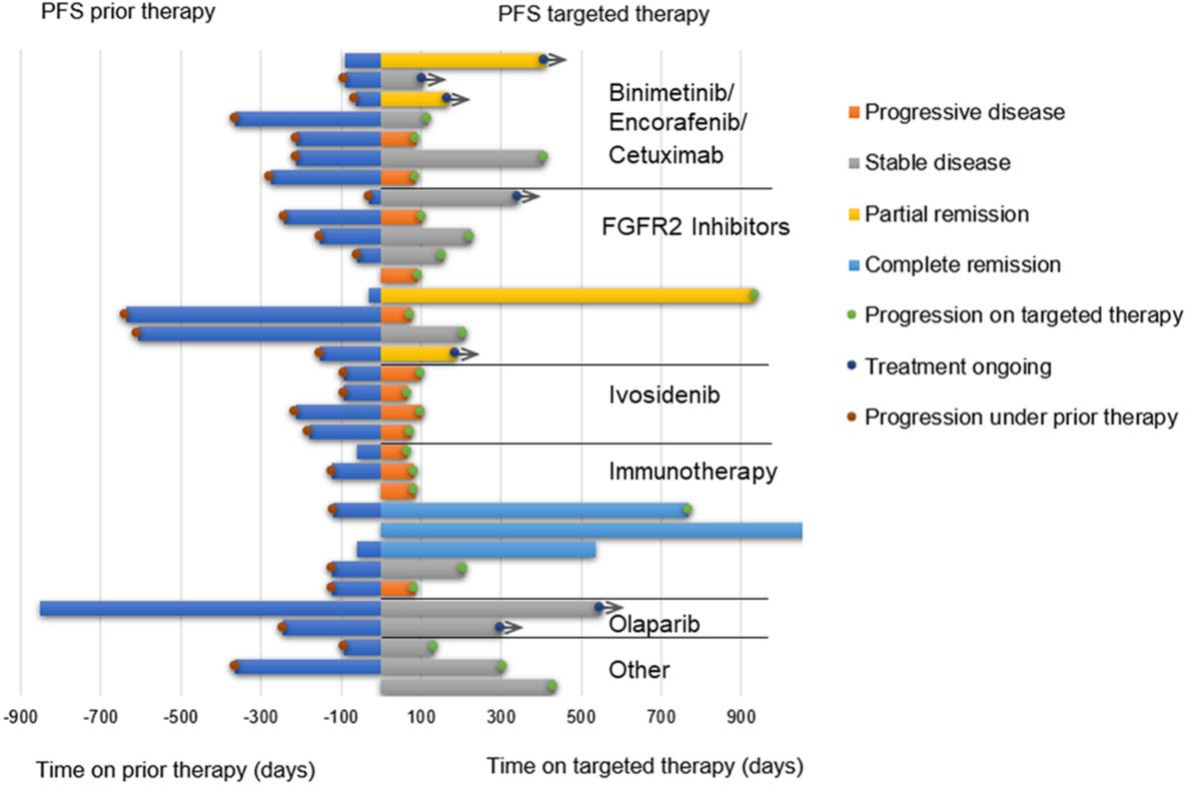
Progression free survival (PFS) under the therapeutic regimen directly preceding the molecular therapies (left) versus PFS under the molecularly targeted therapy (right). Colors indicating the response at 3 months follow-up

**Fig. 6:**
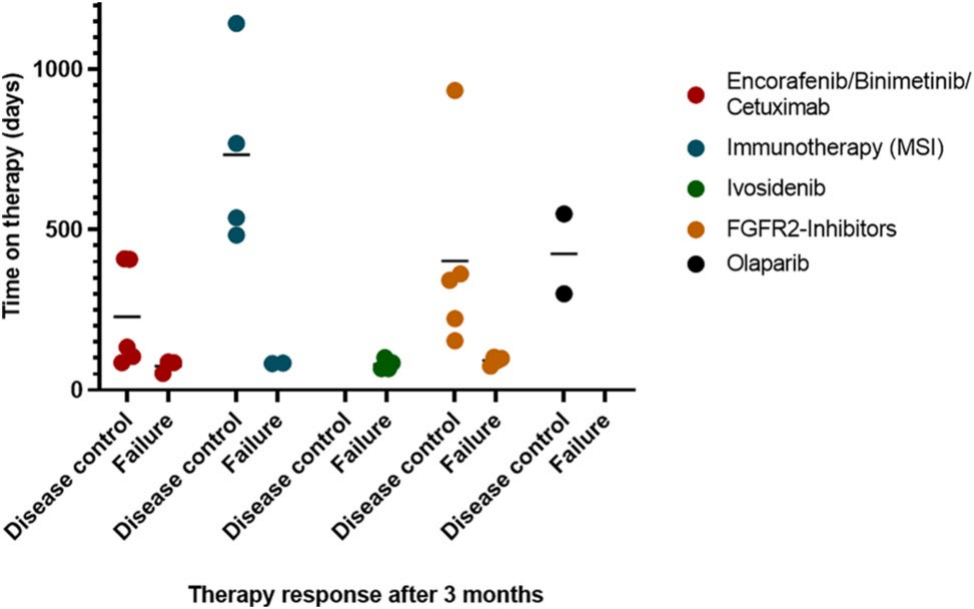
Analysis of therapy duration with subdivision into groups with disease control (patients with complete or partial remission and stable disease) and no response (disease progression) at 3 month follow-up

Individualized treatments based on molecular diagnostics were initiated in 35 patients between March 2019 and April 2020, either following approval by the health insurance company (*n* = 25), or in the context of a compassionate use program (*n* = 10). The most frequent therapies were the combination of cetuximab, encorafenib and binimetinib according to the BEACON trial (*n* = 8), which has by now received EMA approval (Kopetz et al. 2019). Ten patients with intrahepatic cholangiocarcinoma and FGFR2 alterations were treated with an FGFR inhibitor (derazantinib or pemigatinib), with the latter now being approved by EMA in pretreated patients harboring FGFR2 fusions, and five patients received pembrolizumab (Fig. 3d). Follow-up data are available for 33 patients. Two patients with a very high tumor burden at baseline did not reach the 3-month follow-up due to tumor progression. The first imaging was performed after a median time of 11 weeks. The overall response rate was 21.2% with 4 (12.1%) and 3 (9.1%) partial and complete responses, respectively. Disease stabilization was reached as best response in additional 13 patients, resulting in an overall disease control rate of 60.6%. All three patients with a complete response received immunotherapy on the basis of microsatellite instability. At the time of data cutoff, a total of 20 patients had progressed under targeted therapy, of which 13 presented with early progression in the first 3-month follow-up. In patients with disease control, the shortest and longest duration of response or stabilization was 116 and 1143 days, respectively, with a median of 348 days (mean 405 days) (Figs. 5 and 6).

**Fig. 7:**
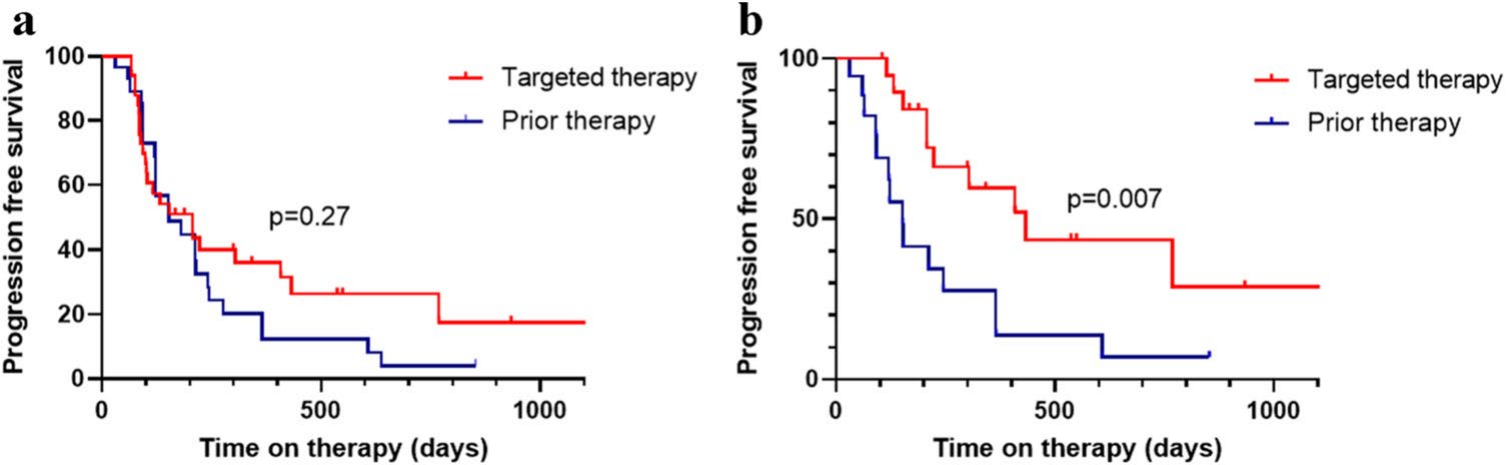
Kaplan-Meier analysis of PFS under molecularly targeted vs. previous therapeutic regimen. **a** all patients with available follow-up data. **b** patients with initial response under targeted therapy

Progression-free survival (PFS) under molecular therapy compared to progression-free survival under the prior treatment regimen was not statistically different but showed a trend in favor of the targeted therapy (Fig. 7a). Furthermore, patients that responded at 3 months after initiation of targeted therapy had a longer progression-free survival under molecular therapy than under the immediate prior treatment regimen (Fig. 7b, *p* value of 0.007), indicating that early response might serve as a surrogate marker for treatment efficacy.

## Discussion

The clinical relevance of precision oncology is increasingly recognized for solid malignancies, including gastrointestinal cancers. Targeted treatments can extend the therapeutic spectrum on an individual patient’s basis and, in some cases, have the potential to significantly alter the clinical course of the disease. Treatment-relevant genetic alterations are frequently diagnosed by panel sequencing. To ensure that efficient therapies are not withheld from the patients, it is critical to choose molecular diagnostics that are capable of detecting all therapeutically relevant genetic alterations.

The selection of a panel is often heavily biased by the diagnostic procedures that are established at the local molecular pathology, and may represent a compromise between cost-effectiveness and diagnostic depth. Some focused panels are customized for specific tumor entities, and may therefore fail to provide sufficiently comprehensive information if applied to other cancers. The importance of matching the panel diagnostics to the genetic landscape of the individual entities is exemplified by biliary tract cancers: while FGFR2 fusions are nearly absent in extrahepatic cholangiocarcinomas, they occur with a high frequency (10–15%) in patients with intrahepatic cholangiocarcinoma (Lamarca et al. 2020). Therefore, although a specific panel might be well suited for diagnostic workup in tumors that arise from the extrahepatic bile ducts, it might fail to detect critical alterations of the intrahepatic counterparts.

An additional and important caveat, which can be easily overlooked by the treating physicians, is, that even if the panel lists specific gene names, not all platforms necessarily cover the entirety of relevant alterations. As an example, a post hoc analysis of a recent clinical trial (FIGHT-202) revealed that only 50% of the detected FGFR2 fusions had been described before (Abou-Alfa et al. 2020b; Silverman et al. 2021). Based on the hybrid capture technology of the Foundation One CDx assay that was used as companion diagnostics within the FIGHT-202 trial, chromosomal FGFR2 rearrangements could be detected without prior knowledge of the partner gene, whereas amplicon-based panels would have failed to detect rearrangements for which specific partner gene primers were missing from the sequencing reaction. The Oncomine Comprehensive Cancer panel v3, for instance, currently only detects 25 different FGFR2 fusions from the > 150 FGFR fusion documented to date. In line with this disparity, we identified FGFR2 fusions in two out of nine patients from our local cohort which had not been detected by the amplicon approach.

Ideally, however, repetitive rounds of sequencing diagnostics should be avoided by choosing the most suitable testing strategies to circumvent unnecessary cost and time loss. To match patients with optimized molecular tests, a close interaction between molecular pathology and the clinical care providers is crucial, and we advocate that these diagnostics should be best performed in centralized referral centers. Traditionally, pathologists were primarily involved in the initial organ-specific classification of the malignant disease. Now, the molecular pathologist is becoming more visible in daily clinical practice, as informed clinical decision making warrants the careful evaluation and interpretation of sequencing results. The importance of this interaction is reflected by the growing number of molecular tumor boards (Hoefflin et al. 2021).

Finally, standardized “tracking” systems for tissues shipped from external pathologies are lacking, and uncertainty concerning the whereabouts of the materials that are needed for panel sequencing can further complicate the diagnostic processes and prolong treatment initiation. Together, this leads to an unacceptable number of patients that deteriorate prior to initiation of targeted therapies.

Our single-center experience illustrates that the integration of precision medicine in clinical treatment concepts continues to be a challenge in GI oncology in Germany: Especially in “rare” malignancies such as cancers of the biliary system, the individual genetically defined subgroups are small, which often hampers patient accrual for precision oncology trials. Positive data from phase III trials, however, are commonly expected before targeted agents gain approval by the European Medical Agency. Prior to EMA approval, physicians are usually required to file for cost coverage by the insurance providers based on the individual clinical records, which can be a time-consuming process. In some cases, the timeframe until a response was issued by the German insurance providers exceeded 5 weeks. Furthermore, the experience of our real-world cohort shows that the reasons for denial of coverage are often not based on a lack of evidence. In multiple cases, applications were rejected despite a high level of evidence (ESCAT IA). Early access/compassionate use programs can fill the gap between clinically meaningful data and EMA approval. In the absence of suitable clinical trials, the possibility to include patients into these programs should be explored.

In our analysis, the comparison of progression-free survival favored the personalized therapy approach, and we observed a significant increase in the progression-free survival under molecular therapy compared to the immediate prior therapy in those patients that initially responded to the personalized approach. To further optimize outcome, and avoid toxicity as well as unnecessary cost of targeted drugs, molecular as well as clinical “biomarkers”, such as optimal timing of response assessment, should be evaluated consistently and assessed for their suitability as early predictors of treatment efficacy. Of note, patients were frequently referred to our center at advanced disease stages, and after several lines of therapy. Therefore, it is well conceivable, that the efficiency of precision oncology in real-world cohorts from GI cancer patients do not meet the responses reported from clinical trials. Especially in cancers with a limited number of effective or approved treatments available, such as cholangiocarcinoma, we strongly advocate the early implementation of molecular diagnostics, even though insurance agencies in Germany usually grant cost coverage for individualized approaches only after exhaustion of standard treatments. This early testing strategy is also endorsed by recommendations published by the European Society of Medical Oncology (ESMO) (Mosele et al. 2020).

In summary, we believe that the potential of precision medicine in GI cancers is not yet being fully exploited in Germany and that the hurdles that need to be taken into account before a patient receives a molecular therapy are substantial and time consuming.

More precise guidelines on the initiation of NGS diagnostics, and routine referral to reference centers with ample experience in the contextual interpretation of and reaction to molecular results, would likely be beneficial in the implementation of precision oncology in Germany.

## Supplementary Information

Below is the link to the electronic supplementary material.

Supplementary file1 (DOCX 34 KB)

## Data Availability

Not applicable.
