# Supplementary material for: Molecular diagnostics and therapies for gastrointestinal tumors: a real-world experience
Source: J Cancer Res Clin Oncol. 2021 Aug 26;148(8):2137–44. doi: 10.1007/s00432-021-03774-5 (PMC9293869; doi:10.1007/s00432-021-03774-5)
Supplement: Supplementary file 1 — Supplementary file1 (DOCX 34 KB) [file 432_2021_3774_MOESM1_ESM.docx]

Supplement

Table 1

|  | All patients with actionable lesions (n=53) | Patients on targeted treatment (n=35) |
| --- | --- | --- |
| *Median Age at diagnosis (range)* | ***57.5 (18-89)*** | ***60 (36-89)*** |
| *Gender, n (%)* |  |  |
| Male | 31 (58) |  |
| Female | 22 (42) |  |
| *Primary resection, n (%)* | ***26 (47)*** | ***22 (62.9)*** |
| CCC/Gallbladder cancer | 11 | 7 |
| Colorectal cancer | 10 | 10 |
| Gastric- /AEG cancer | 3 | 3 |
| Others | 2 | 2 |
| Neoadjuvant treatment | 1 | 1 |
| Adjuvant treatment | 15 | 11 |
| UICC stage at initial diagnosis |  |  |
| UICC stage I | 4 (7.5) | 4 (11.4) |
| UICC stage II | 6 (11.3) | 4 (11.4) |
| UICC stage III | 12 (22.7) | 8 (22.9) |
| UICC stage IV | 29 (54.8) | 18 (51.4) |
| No data available | 2 (3.7) | 1 (2.9) |
| Therapy lines at time of application (mean) | 2.75 | 2.76 |
| 1^st^ line therapy, n (%) | 45 (84.9) | 32 (94.3) |
| mPFS 1^st^ line (months) | 4.48 | 5.1 |
| 2^nd^ line therapy, n (%) | 28 (52.8) | 20 (57.1) |
| mPFS 2^nd^ line (months) | 3.03 | 4.06 |
| 3^rd^ line therapy, n (%) | 11 (20.8) | 10 (28.6) |
| mPFS 3^rd^ line (months) | 3.48 | 4.0 |
| Median time from first diagnosis to start targeted therapy (months) | 18.05 | 19 |

Baseline characteristics of 53 patients with identified target for treatment and 35 patients started on targeted treatment.

Table 2

| Applied therapy | Clinical Trial | ESCAT-level | Insurance response |
| --- | --- | --- | --- |
|  |  |  |  |
| BRAF Inhibition  in CRC (n=13) in CCC (n=2)  in PDCA (n=1) | BEACON-Trial, Phase III (Van Cutsem et al., 2019)  ClinicalTrial NCT01524978, Phase II (Hyman et al., 2015) ROAR-Trial, Phase II (Hyman et al., 2015; Subbiah et al., 2020)  ROAR-Trial, Phase II (Hyman et al., 2015; Subbiah et al., 2020) | IA  IIB  IIB  IIIA | 9/ 1/ 2/ 1  1/ 0/ 1/ 0  1/ 0/ 0/ 0 |
| Immunotherapy in CRC with MSI-H (n=4)  in GC with  MSI-H (n=3)  in CCC with MSI-H (n=1)  in PDAC  MSI-H (n=1) | Keynote -164, Phase III (Janjigian et al., 2018)  CheckMate 142, Phase II (Overman et al., 2017)  CheckMate -032 (Janjigian et al., 2018)  Keynote-158, Phase II (Marabelle et al., 2020)  Keynote-158, Phase II (Marabelle et al., 2020) | IA  IC  IC  IC  IC | 1/ 1/ 1/ 1  1/ 1/ 1/ 0  1/ 0/ 0/ 0  1/ 0/ 0/ 0 |
| Immunotherapy  in CPS-positive GC (n=5) | CheckMate -649, Phase III (Pietrantonio et al., 2021)  Keynote-062, Phase III (Pietrantonio et al., 2021) | IA | 3/ 0/ 0/ 2 |
| IDH1-Inhibition  in CCC (n=4) | ClarIDHY-Trial, Phase III (Abou-Alfa et al., 2020) | IA | 4/ 0/ 0/ 0 |
| FGFR2 Inhibition | FIGHT-202, Phase-II (Abou-Alfa et al., 2020)  Phase I/II Derazantinib (Busset et al., 2019)  Phase III Futibatinib (Bridgewater et al., 2020)  BGJ398 Phase-II Infigratinib (Javle et al., 2021) | IB | 10 patients in EAP |
| BRCA Inhibition  in PDCA (n=2)  in GBCA (n=1)  in GC (n=1) | POLO-Trial, Phase III (Golan et al., 2019)  POLO-Trial, Phase III (Golan et al., 2019)  POLO-Trial, Phase III (Golan et al., 2019) | IA  IIIA  IIIA | 2/ 0/ 0/ 0  0/ 0/ 1/ 0  0/ 0/ 0/ 1 |
| ERBB2 Inhibition in CRC (n=1)  in CCC (n=1) | MyPathway-Trial, Phase II (Meric-Bernstam et al., 2019) HERACLES-Trial, Phase II (Sartore-Bianchi et al., 2016)  MyPathway-Trial, Phase II (Meric-Bernstam et al., 2019) | IIB  IIA  IIB | 1/ 0/ 0/ 0  0/ 0/ 1/ 0 |
| Immunotherapy in SCC  Anal canal (n=2)  Esophagus (n=1) | ClinicalTrials.gov NCT02314169, Phase II (Morris et al., 2017)  Keynote -028; Phase II (Ott et al., 2017)  ATTRACTION -3, Phase III (Kato et al., 2019)  Keynote -181, Phase III (Kojima et al., 2020) | IIB  IA | 0/ 0/ 2/ 0  1/ 0/ 0/ 0 |
|  |  |  | 26/3/9/5 |

Summary of cost coverage requests filed to the health insurance providers. Column A: Molecular target and tumor entity. Column B: studies referred to in application. Column C: ESCAT evidence level. Column D: Response of the health insurance responses: number of approvals/ number of approvals after initial rejection/ rejection/ number of patients deceased before approval.

Abbreviations: GC: Gastric cancer, PDCA: pancreatic ductal adenocarcinoma, CCA: Cholangiocellular carcinoma, CRC: Colorectal carcinoma, SCC: Squamous cell carcinoma, GBCA: Gallbladder carcinoma
